# Beyond licensure: systemic reforms to expand evidence-based addiction treatment

**DOI:** 10.1093/haschl/qxaf086

**Published:** 2025-04-18

**Authors:** David T Zhu, Suhanee Mitragotri

**Affiliations:** Medical Scientist Training Program, School of Medicine, Virginia Commonwealth University, Richmond, VA 23298, United States; Department of Molecular & Cellular Biology, Harvard University, Cambridge, MA 02138, United States

Busch et al.'s recent cross-sectional analysis identifies persistent gaps in the availability of medications for opioid and alcohol use disorders (MOUD and MAUD) in the United States.^1^ Using data from the 2022 National Substance Use and Mental Health Services Survey, the authors found that among 9921 outpatient behavioral health programs, only about half provided MOUD (51%) and even fewer offered MAUD (45%). Notably, national accreditation was positively associated with medication availability, while state licensure or certification showed a negative association. This likely reflects their differing purposes: state licensure is mandatory and typically ensures compliance with minimum safety, staffing, and regulatory standards, whereas national accreditation, offered by organizations such as The Joint Commission, focuses on care quality, clinical protocols, and use of evidence-based practices.^1^

Currently, only a few states require outpatient treatment programs to offer MOUD/MAUD as a condition of licensure. Louisiana, Missouri, and New York mandate that programs provide MOUD on-site or facilitate access through referral.^2^ California and Massachusetts prohibit programs from denying admission to individuals already receiving these medications.^2^ Incorporating MOUD/MAUD into licensure requirements could, in theory, expand access to populations underserved by Medicaid policy. Medicaid regulations apply only to participating providers, whereas state licensure applies to all facilities within a state, regardless of payer mix, potentially offering a more universal regulatory lever.^2^

However, mandating MOUD/MAUD through licensure raises legitimate concerns. Programs lacking adequate clinical infrastructure or financial capacity may be forced to close, particularly in resource-constrained settings.^3^ Smaller or grant-funded programs may also struggle to absorb any additional costs without technical assistance or reimbursement support. Workforce shortages, particularly of qualified prescribers, remain another major barrier.^4^ To inform policy, future research should evaluate the effects of licensure mandates on treatment access, retention, and overdose outcomes, and to assess the overall feasibility of incorporating MOUD/MAUD into licensure requirements.

Furthermore, expanding licensure mandates alone is unlikely to suffice, as it is important that policy reforms are implemented alongside operational infrastructure, provider support, and sustainable financing. For instance, Vermont's hub-and-spoke system, launched in 2013, integrates office-based buprenorphine treatment (“spokes”) with regional specialty centers (“hubs”) that provide methadone and complex care, by which the state achieved a 64% increase in buprenorphine-waivered physicians and a 50% increase in patients served per prescriber.^5^ As states weigh licensure-based mandates, their effectiveness will hinge on building robust clinical and public health infrastructure to support implementation and ensure continuity of care.

## Contribution statement

D.T.Z. and S.M. conceptualized, wrote, and revised the manuscript.

## Supplementary Material

qxaf086_Supplementary_Data
